# Induction of Embryogenic Callus, Protoplast Isolation, and PEG-Mediated Transformation Protocols in *Eucommia ulmoides*

**DOI:** 10.3390/plants15020194

**Published:** 2026-01-08

**Authors:** Hongrun Zhou, Zibo Zhou, Jiangyuan Zhang, Haoran Kan, Mengqi Yin, Han Zhang, Luyao Wang, Jie Zhao, Jing Ye

**Affiliations:** 1 Laboratory of Forestry Department, Agricultural College, Shihezi University, Shihezi 832003, China; zhouhongrun@stu.shzu.edu.cn (H.Z.); zhouzibo9308@outlook.com (Z.Z.); yinmengqi0623@163.com (M.Y.); zhanghanfo@foxmail.com (H.Z.); wangluyao0806@foxmail.com (L.W.); 2 Key Laboratory of Xinjiang for Oasis Agricultural Pests Management and Plant Protection Resources Utilization, Agriculture College, Shihezi University, Shihezi 832003, China; zhangjiangyuanDA@163.com (J.Z.); kanhaoran@stu.shzu.edu.cn (H.K.)

**Keywords:** *Eucommia ulmoides*, embryogenic callus, protoplast isolation, transient transformation

## Abstract

*Eucommia ulmoides*, a tree species native to China, holds considerable medicinal, ecological, and industrial importance. However, the absence of an efficient and stable genetic transformation system poses significant challenges to gene function studies and molecular breeding in *E. ulmoides*. Protoplasts, which lack cell walls, serve as effective receptors for transient transformation and are thus ideal for genetic engineering research. In this study, the optimal conditions for callus induction were identified, and formation of the embryogenic callus was confirmed by histological analysis. Furthermore, we developed an efficient protoplast isolation and PEG-mediated transient transformation system using suitable embryogenic callus as the starting material. Our findings revealed that the optimal medium for inducing embryogenic callus was B5 + 1.5 mg/L 6-BA + 0.5 mg/L NAA + 30 g/L sucrose + 7 g/L agar (pH = 5.8). In this medium, the induction rate of callus achieved 97.50%, and the rate of embryogenic callus formation was 86.30%. For protoplast isolation, the best conditions involved enzymatic digestion with 1.5% cellulase R-10 and 1.0% macerozyme R-10 at an osmotic pressure of 0.6 M for 4 h, resulting in 1.82 × 10^6^ protoplasts/g FW with 91.13% viability. The highest transfection efficiency (53.23%) was attained when protoplasts were cultured with 10 µg of plasmid and 40% PEG4000 for 20 min. This study successfully established a stable and efficient system for protoplast isolation and transient transformation in *E. ulmoides*, offering technical support for exploring somatic hybridisation and transient gene expression in this species.

## 1. Introduction

*Eucommia ulmoides* Oliv., a distinctive economic tree species native to China, holds considerable medicinal, ecological, and industrial significance [1,2]. The bark and leaves of *E. ulmoides* are abundant in bioactive compounds like iridoids, lignans, and flavonoids, which have impressive pharmacological properties, including anti-inflammatory effects, blood pressure regulation, and bone metabolism modulation [3,4,5]. In the industrial sector, *Eu*-rubber stands out as a unique bio-based polymer resource in China, emerging as a natural polymer material. Predominantly found in the bark, stems, leaves, seeds, and other tissues of *E. ulmoides* [6,7], this polymer offers a strategic alternative to natural rubber [3,8]. Furthermore, *E. ulmoides* demonstrates robust tolerance to abiotic stresses such as cold and drought, making it a valuable model for exploring stress resistance mechanisms and secondary metabolism regulation in woody plants [9,10,11].

Plant protoplasts, which are plant cells stripped of their cell walls through enzymatic processes, retain their regenerative ability, viability, and metabolic activity [12]. They are crucial for plant fundamental research and serve as invaluable tools for enhancing crop genetics [13]. Protoplasts can be isolated from various plant tissues, such as leaves, cotyledons, petals, roots, hypocotyls, suspension-cultured cells, and callus [14]. Notably, using actively growing and young tissues for isolation results in protoplasts with higher yields and improved viability [15]. Plant embryogenic callus, characterised by dense cytoplasm, prominent nuclei and active cell division, is particularly well-suited for protoplast isolation [16]. Furthermore, embryogenic callus is preferable for enzymatic digestion due to its relatively thin cell walls and low levels of lignification and secondary metabolites like polyphenols and polysaccharides, resulting in a higher yield and viability of protoplasts compared to non-embryogenic callus [17,18,19,20]. The protoplasts derived from embryogenic callus are more apt for transient transformation and gene function studies, laying the groundwork for plant regeneration and genetic transformation research [21]. Consequently, this study selected embryogenic callus as the source material for protoplast isolation, with systematic optimisation of the isolation conditions.

Plant protoplasts offer a versatile tool for studying gene functions, such as subcellular localisation, protein interactions, and genome editing, and can also act as intermediaries in generating stable transgenic plants [22,23]. By employing transient plasmid transfection, protoplasts can swiftly express foreign genes, facilitating rapid functional verification. Given that Agrobacterium-mediated stable transformation is expensive, time-intensive, and technically challenging, the protoplast transient transfection system presents a rapid, adaptable, and cost-effective alternative for plant molecular research [24,25,26]. Furthermore, protoplasts serve as efficient explants for quick gene function assays, as transfected plasmids are promptly expressed within the cells, allowing for immediate evaluation of gene activity. Subcellular localisation analysis, which identifies the intracellular distribution of gene products, is a prevalent method for functional verification [27,28,29]. Transient transfection systems have been successfully implemented in various plant species, including *Arabidopsis* [30,31], rice [14], maize [32], wheat [33,34], soybean [35,36], and strawberry [37], to facilitate subcellular localisation studies. Previous studies on woody plants such as *Cunninghamia lanceolata* [38], poplar [39,40], grape [41,42], citrus [27,43], walnut [23], and *Camellia oleifera* [44,45,46] have shown that optimising enzyme composition, digestion time, osmotic pressure, and pretreatment conditions can significantly improve protoplast yield and viability. These findings provide valuable insights for developing efficient protoplast systems in woody species.

In woody species, the thick cell walls and high concentrations of secondary metabolites, such as polyphenols and polysaccharides, render cells susceptible to damage and complicate the maintenance of viability during protoplast isolation. As a result, yields tend to be low, and a robust isolation system remains elusive. Based on the aforementioned research background, this study seeks to achieve a high yield and viability of protoplasts derived from the embryogenic callus of *E*. *ulmoides*, while also establishing an efficient protoplast transient transformation system. Initially, the hypocotyl was utilised as an explant to investigate the impact of varying concentrations of 6-BA and NAA on the callus induction rate, alongside a microscopic examination of tissue sections to elucidate the embryogenic callus type. Subsequently, employing the embryogenic callus as the substrate, different enzyme concentrations, digestion durations, and centrifugation speeds were investigated to ascertain the optimal conditions for isolating and purifying protoplasts. Lastly, the influence of various plasmid and PEG4000 concentrations, along with transformation durations, on protoplast transformation efficiency was evaluated to establish a proficient protoplast transient transformation system. This study can offer robust technical support for somatic hybridisation and fusion, as well as for the subcellular localisation and transient expression of functional genes in *E. ulmoides*, providing valuable resources for functional genomics research and molecular breeding within this species.

## 2. Results

### 2.1. Embryogenic Callus Induction and Cytological Identification

**Figure 1:**
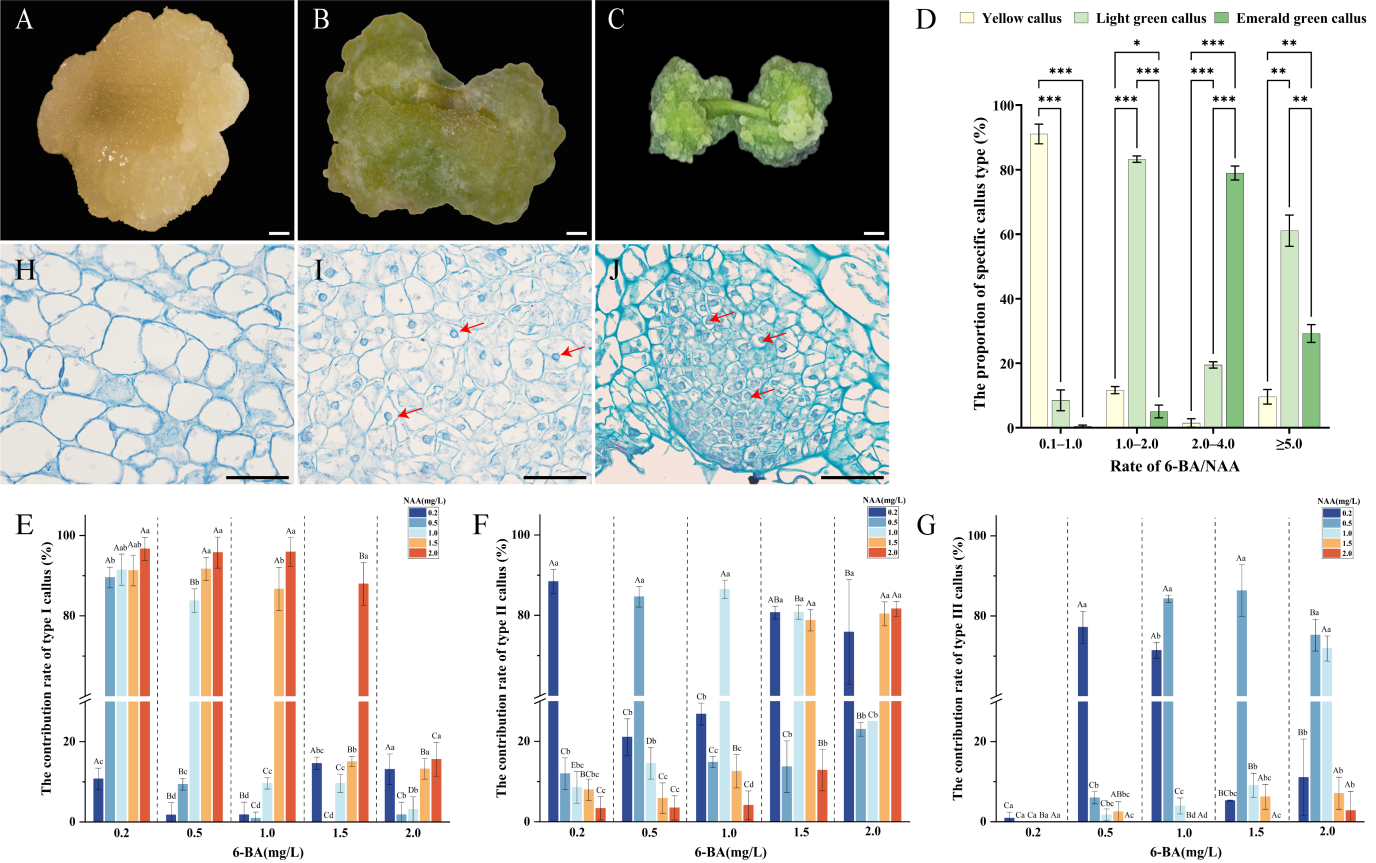
Effect of hormone ratios on callus type induction and morphological and cytological observations of callus in *E. ulmoides*. (**A**) Type I callus, scale bar = 1 mm (the same for (**B**,**C**)); (**B**) type II callus; (**C**) type III callus; (**D**) the proportion of three callus types under varying 6-BA/NAA ratios, in which “*”, “**”, “***” denote significant differences at *p* < 0.05, *p* < 0.01, and *p* < 0.001, respectively. (**E**–**G**) Effects of different 6-BA and NAA concentration combinations on the contribution rates of type I, II, and III callus, different uppercase letters indicate significant differences among different 6-BA concentrations at the same NAA concentration (*p* < 0.05), while different lowercase letters indicate significant differences among different NAA concentrations at the same 6-BA concentration (*p* < 0.05). (**H**–**J**) Histological observations of type I, II, and III callus under a light microscope, scale bar = 100 µm, red arrows highlight the nuclei.

A two-factor, five-level full factorial L25 experimental design was utilised to formulate media for callus induction from the hypocotyl. The results indicated that all 25 treatments successfully induced callus, with induction rates exceeding 80% in every instance. Remarkably, 12 combinations achieved induction rates above 95%, and two treatments reached a perfect 100% induction rate (Table 1). Analysis of variance revealed that both the main effects and the interaction between 6-BA and NAA concentrations on the callus induction rate were highly significant (*p* < 0.001), with 6-BA exerting the most substantial influence (F = 69.969, *p* < 0.001). Generally, low concentrations of 6-BA, when paired with appropriate NAA levels, enhanced callus induction rates. In contrast, at high 6-BA concentrations, increasing NAA was inhibitory. During the formation of callus in *E*. *ulmoides*, the colour and texture of explants undergo significant changes. In type I, the explants exhibited a yellow color, no obvious protrusions, and a loose texture with a water-soaked appearance (Figure 1A). In type II, the colour shifted to light green, and the texture became dry and firm with no discernible protrusions (Figure 1B). In type III, the explants turned bright green and maintained a dry and firm texture with spherical protrusions (Figure 1C). The results indicated that various combinations of 6-BA and NAA concentrations significantly influence the state of callus on the 30th day.

The ratio of cytokinin to auxin is crucial in regulating callus formation, prompting an analysis of the effect of 6-BA/NAA ratio on callus induction rates. The findings revealed significant variations in the proportion of three callus types under different 6-BA/NAA ratios (Figure 1D). At a ratio of 0.1–1.0, it was mostly type I callus, comprising 91.1%, while type II and type III callus were lower, at 8.5% and 0.4%, respectively. When the ratio increased to 1.0–2.0, type II callus became predominant at 83.3%, with type I and type III at 11.6% and 5.1%, respectively. A ratio of 2.0–4.0 primarily was type III callus (79.0%), while type II and type I were less frequent at 19.5% and 1.5%, respectively. When the ratio exceeded 5.0, the proportions of the three callus types were 9.7% for type I, 61.1% for type II, and 29.2% for type III. Subsequently, the contribution rate of each medium to the three callus types was analysed further. Type I callus was most prevalent in the combination of low-concentration 6-BA (0.2–1.0 mg/L) and high-concentration NAA (1.5–2.0 mg/L), with the highest contribution rate of 96.7% achieved at 0.2 mg/L 6-BA + 2.0 mg/L NAA (Figure 1E). Type II callus primarily depended on the ratio of 6-BA and NAA, exhibiting the highest quantity at a ratio of 1.0, with a maximum contribution rate of 88.4% observed in the medium containing 0.2 mg/L 6-BA and 0.2 mg/L NAA (Figure 1F). Type III callus required a 6-BA concentration of 0.5 mg/L or higher, and a relatively low NAA concentration (0.2–0.5 mg/L). The highest contribution rate of type III was 86.3% with 1.5 mg/L 6-BA + 0.5 mg/L NAA (Figure 1G).

To accurately identify embryonic callus for protoplast isolation, the three callus types were processed into permanent paraffin sections for histological observation. The analysis revealed significant differences in their cellular structures. The cells of type I callus were varied in size, irregularly shaped, loosely arranged, and had fewer nuclei, which exhibited the characteristic of non-embryogenic callus (Figure 1H). The cells of type II callus were more uniform, regularly shaped, relatively compact, and clearly visible nuclei, which exhibited the characteristics of embryogenic callus (Figure 1I). However, the nucleo-cytoplasmic ratio of type II callus is relatively low, making it unsuitable as a material for protoplasts. In contrast, type III callus cells were densely packed, orderly, with large, distinct nuclei and a high nucleo-cytoplasmic ratio, which was typical of embryogenic callus and more suitable for protoplasts (Figure 1J).

Consequently, hypocotyls from 15-day-old sterile *E. ulmoides* seedlings served as explants. And cultured on a medium comprising B5 + 1.5 mg/L 6-BA + 0.5 mg/L NAA + 30 g/L sucrose + 7 g/L agar, (pH = 5.8), for 30 days. The resulting emerald green callus was then utilised for protoplast isolation.

### 2.2. Optimisation of Protoplast Isolation System from Embryogenic Callus

Based on 30-day embryogenic callus, different concentrations of cellulase R-10 and macrozyme R-10, and various enzyme digestion times were set to evaluate their effects on protoplast yield and viability. The range analysis reveals that these three factors exert differing levels of influence on both yield and viability. Notably, the concentration of cellulase R-10 significantly affected protoplast yield, showing the highest impact (F = 746.046, *p* < 0.001, R = 0.957). This was followed by enzymolysis time (F = 14.426, *p*< 0.001, R = 0.130), with macrozyme R-10 having the least effect (F = 8.046, *p* < 0.001, R = 0.089). Conversely, for protoplast viability, macrozyme R-10 concentration was the most influential factor (F = 124.429, *p* < 0.001, R = 6.88), followed by cellulase R-10 concentration (F = 60.013, *p* < 0.001, R = 3.82), while enzymolysis time had the least impact (F = 0.718, *p* = 0.547, R = 0.50).

**Figure 2:**
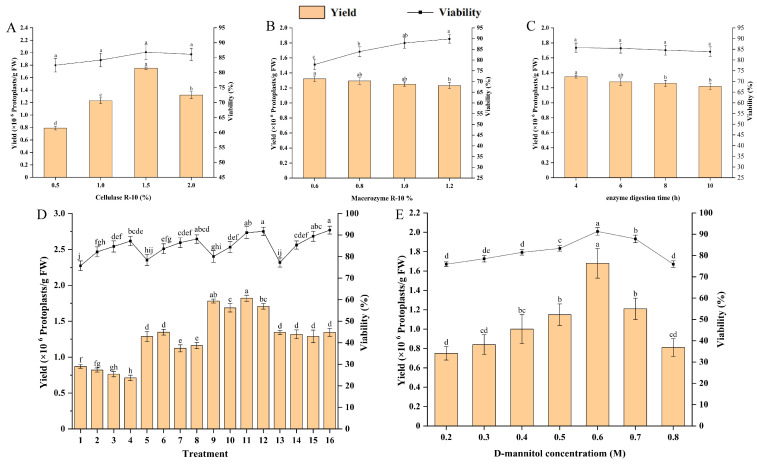
Optimisation of protoplast isolation system derived from embryogenic callus in *E. ulmoides*. (**A**) Effects of cellulase R-10 concentration on protoplast yield and viability; (**B**) effects of macrozyme R-10 concentration on protoplast yield and viability; (**C**) effect of enzymolysis time on protoplast yield and activity; (**D**) the orthogonal experiment of cellulase R-10 and macerozyme R-10 concentrations as well as enzymolysis time on protoplasts yield and viability; (**E**) effects of D-Mannitol concentration on protoplast yield and viability. The different lowercase letters indicate significant differences (*p* < 0.05).

To identify the optimal levels for protoplast isolation factors, we conducted an LSD multiple comparison analysis on yield and viability at each factor level (Figure 2). The finding revealed that a cellulase R-10 concentration of 1.5% yielded the highest protoplast yield and viability (Figure 2A). Interestingly, as the macerozyme R-10 concentration increased, protoplast yield decreased, while viability improved (Figure 2B). Balancing both yield and viability, we determined that a macerozyme R-10 concentration of 1.0% was optimal. Prolonged enzymolysis time led to reduced yield and viability (Figure 2C), indicating that 4 h is the optimal enzymolysis duration. In addition, we performed an orthogonal experiment to comprehensively evaluate the effects of different concentrations of cellulase R-10 and macrozyme R-10, as well as enzymolysis time, on protoplast yield and viability. The findings showed a significant increase in yield up to treatment 11, followed by a sharp decline. Protoplast activity was highest in treatments 16 and 12, standing at 92.30% and 91.73%, respectively, but treatment 11 also exhibited a high level of 91.13%, and there was no significant difference among these treatments (Figure 2D). Consequently, we concluded that treatment 11 offers the optimal conditions for protoplast isolation, involving enzymolysis for 4 h with a combination of 1.5% cellulase R-10 and 1.0% macerozyme R-10.

We examined the influence of D-mannitol concentration as an osmotic regulator on protoplast yield and viability. The concentration of D-mannitol significantly affected both yield and viability (*p* < 0.05). As the D-mannitol concentration increased, protoplast yield and viability improved, peaking at 0.6 M. Beyond this concentration, both yield and viability notably declined (Figure 2E). Thus, 0.6 M D-mannitol was identified as the optimal concentration for protoplast isolation.

### 2.3. Optimisation of Protoplast Purification Conditions

**Figure 3:**
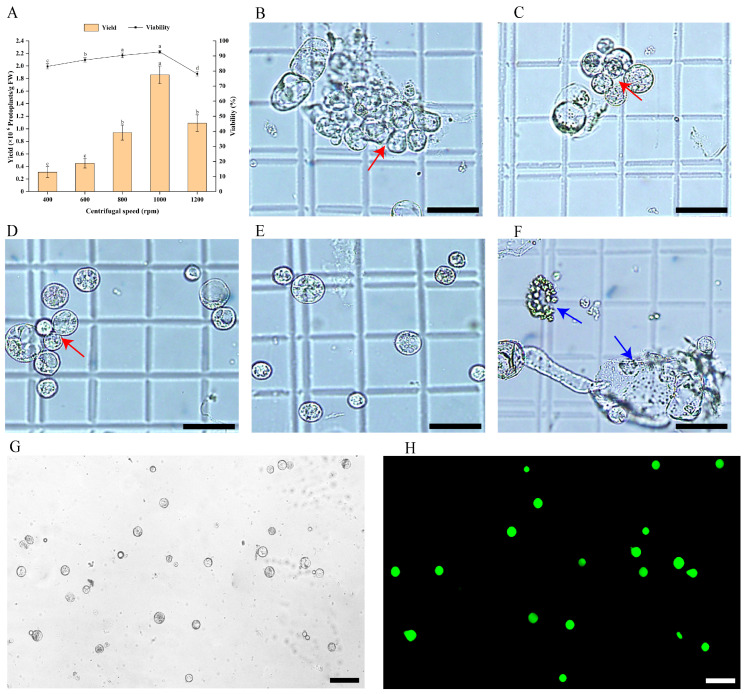
Effect of centrifugal speed on protoplast purification in *E. ulmoides*. (**A**) Protoplast yield and viability at different centrifugal speeds, in which different lowercase letters indicate significant differences (*p* < 0.05). (**B**) Microscopic morphology of protoplasts at 400 rpm, scale bar = 50 µm (the same as (**C**–**F**)). (**C**) Microscopic morphology of protoplasts at 600 rpm. (**D**) Microscopic morphology of protoplasts at 800 rpm. (**E**) Microscopic morphology of protoplasts at 1000 rpm. (**F**) Microscopic morphology of protoplasts at 1200 rpm. (**G**,**H**) Observation of the morphology of protoplasts under bright field and 488 nm excitation light using a fluorescence microscope after FDA staining, scale bar = 75 µm. Notes: Red arrows indicate aggregated protoplasts (in (**B**–**D**)). The blue arrow represents the damaged protoplast (in (**F**)).

To collect protoplasts and eliminate impurities such as non-enzymolysis substances, cell debris, and shrunken cells, the enzymatic mixture was filtered twice through a 70 μm cell sieve and subsequently centrifuged at varying speeds. The results indicated that centrifugal speed significantly affected both the yield and viability of protoplasts (*p* < 0.05), as well as their morphology (Figure 3). At 400 rpm, the protoplast yield was minimal, viability was poor, and most protoplasts clustered together (Figure 3A,B). Similarly, at 600 rpm, yield and viability remained low, with many protoplasts interconnected (Figure 3A,C). However, at 800 rpm, both yield and viability improved markedly, although the protoplasts were unevenly dispersed in the W5 solution (Figure 3A,D). The optimal results were achieved at 1000 rpm, yielding the highest protoplast count (1.82 × 10^6^ protoplasts/g FW) and viability (92.13%), with protoplasts exhibiting complete morphology and even distribution (Figure 3A,E). Post-FDA staining, they displayed strong fluorescence and uniform staining, indicating high vitality and good physiological condition (Figure 3G,H). Conversely, increasing the speed to 1200 rpm resulted in a significant decline in yield and viability, with many protoplasts rupturing due to excessive centrifugal force (Figure 3A,F). Consequently, 1000 rpm was identified as the optimal centrifugation speed for protoplast purification.

**Figure 4:**
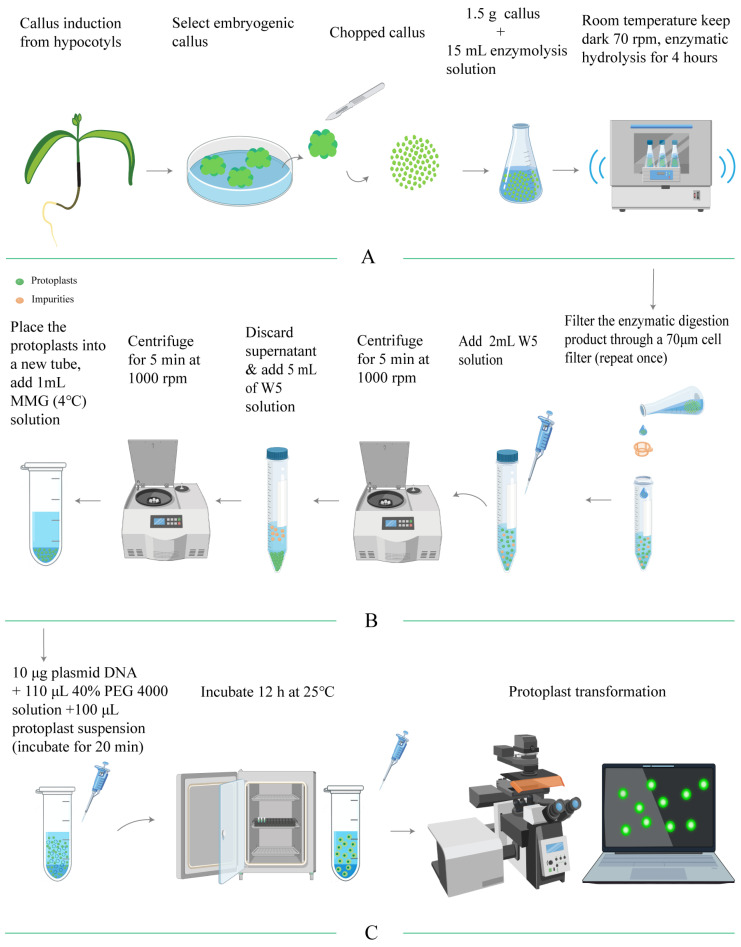
Flowchart detailing the processes of protoplast isolation, purification, and transient transformation in *E. ulmoides*. (**A**) outlines the method for isolating protoplasts; (**B**) describes the purification process; (**C**) presents the method for transient transformation of protoplasts.

In conclusion, we have established an experimental protocol for isolating and purifying protoplasts from the embryogenic callus of *E. ulmoides*, which is illustrated in a flowchart (Figure 4). According to this protocol, protoplasts with a yield of 1.82 × 10^6^ protoplasts/g FW and a viability of 91.13% could be obtained.

### 2.4. Optimisation of Protoplast Transient Transformation System

**Figure 5:**
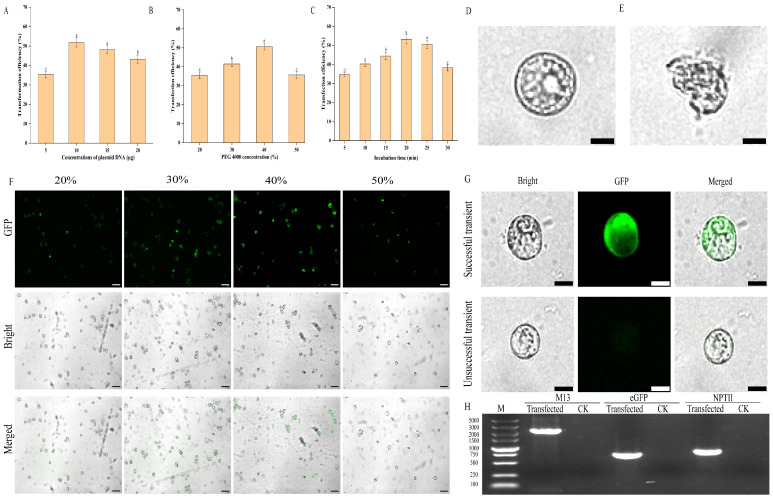
Effects of various factors on protoplast transformation efficiency in *E. ulmoides*. (**A**) The effect of plasmid concentration on transformation efficiency. (**B**) The effect of PEG4000 concentration on conversion efficiency. (**C**) The effect of incubation time on transformation efficiency. (**D**) Protoplasts with complete structure, scale bar = 20 µm (the same for (**E**,**G**)). (**E**) Morphologically damaged protoplasts. (**F**) Observation of protoplast transformation under different PEG4000 concentrations by fluorescence microscope, scale bar = 100 µm. (**G**) Observation of successfully and unsuccessfully transformed protoplasts by fluorescence microscopy. (**H**) Detection of the presence of pCAMBIA3301 plasmid fragment, *eGFP* and *NPT II* in protoplasts transformed with pCAMBIA3301-35S-eGFP by PCR, in which CK represents protoplasts without transformation. Note: different lowercase letters indicate significant differences (*p* < 0.05) in (**A**–**C**).

To develop an efficient protoplast transient transformation system for *E. ulmoides*, we assessed the influence of plasmid concentration, PEG4000 concentration, and transformation duration on transformation efficiency. Our findings revealed that the optimal transformation efficiency (51.87%) was achieved with a plasmid concentration of 10 μg. Variations in plasmid concentration, either higher or lower, significantly reduced the transformation rate (Figure 5A), and excessive plasmid levels caused visible damage to protoplasts (Figure 5D,E). Furthermore, PEG4000 concentration markedly affected the transformation rate. The optimal transformation rate of 50.63% was observed at a PEG4000 concentration of 40% (Figure 5B). Lower PEG4000 concentrations were not favourable for transient transformation, whereas higher concentrations increased protoplast rupture, thereby diminishing the transformation rate (Figure 5F). Regarding transformation time, efficiency improved with increased duration, peaking at 20 min with a rate of 53.23%. Insufficient transformation time led to inadequate contact between the plasmid and protoplasts, resulting in incomplete transformation. Conversely, extending the duration beyond 20 min caused protoplast damage, reducing the transformation rate (Figure 5C). In summary, the optimal conditions for protoplast transient transformation in *E. ulmoides* involve using 10 μg of plasmid with 40% PEG4000 for 20 min, achieving a transformation rate of 53.23%. The transformation process is illustrated in Figure 4C.

The protoplasts transformed with the pCAMBIA3301-35S-eGFP plasmid maintained their regular morphology and intact cell membranes when observed under a bright-field microscope. Upon excitation at 488 nm, regions expressing the *eGFP* protein emitted a distinct green fluorescence, whereas the untransformed protoplasts showed no autofluorescence (Figure 5G). Additionally, PCR analysis confirmed the presence of the pCAMBIA3301 plasmid fragment and the *eGFP* and *NPTII* genes in the transformed protoplasts, but not in the untransfected ones (Figure 5H). These results indicated that pCAMBIA3301-35S-eGFP plasmid had been successfully introduced into protoplasts, resulting in transient expression.

## 3. Discussion

*E. ulmoides* is a valuable woody plant with notable medicinal and industrial applications [47]. Due to its slow growth, research on the regulation and production of its natural metabolites, functional genes, and molecular breeding primarily relies on plant tissue culture techniques [48,49]. Since 1980, numerous studies on tissue culture of *E. ulmoides* have been reported, investigating various parts such as hypocotyls, cotyledons, leaves, bud-bearing stem segments, and both mature and immature embryos [50,51]. Currently, the exploration and validation of functional genes in *E. ulmoides* predominantly utilise *Agrobacterium*-mediated transformation with hypocotyls as explants. However, the incomplete transgenic regeneration system significantly limits the application of functional genes and molecular breeding research [52,53]. Plant protoplasts are living cells without cell walls, can directly absorb exogenous DNA and undergo somatic hybridisation, making them widely used for genetic modification and improvement in plants [54]. Embryogenic callus is generally regarded as an excellent material for protoplast isolation and culture, forming a crucial foundation for developing an efficient protoplast isolation system [46]. Consequently, this study established a system for inducing embryogenic callus from hypocotyls, isolating protoplasts, and implementing PEG-mediated transient transformation in *E. ulmoides*. This provides a reliable platform for the rapid analysis of gene function and genetic manipulation.

Previous research primarily utilised MS medium combined with 6-BA and NAA to induce callus formation and proliferation in *E. ulmoides* [50,51]. Wang et al. [55] discovered that B5 medium significantly outperformed MS medium concerning callus growth, average budding rate, and positive bud ratio post-transformation. Therefore, this study employed hypocotyls as explants to examine the effects of varying concentrations of 6-BA and NAA in solid B5 medium on the callus type and embryogenic potential of *E. ulmoides*. The results showed that all 25 media combinations produced three distinct callus states, differing in colour, shape, and texture (Figure 1A–C). Traditionally, callus embryogenicity was assessed solely based on external characteristics such as colour, shape, and texture [56,57], which posed observational challenges. To address this, we conducted histological observations using the paraffin section technique. In this study, both type II and type III callus exhibited clearly observable nuclei, indicative of embryogenic cells (Figure 1I,J). However, the type III callus has a dense cytoplasm to form a higher nucleo-cytoplasmic ratio, suggesting superior potential for protoplast isolation and purification (Figure 1J). The external morphology of type III callus was described as “bright green, dry and firm texture with spherical protrusions”, aligning with descriptions in existing literature [58,59]. In this study, the average callus induction rates for 12 culture media exceeded 95%. The concentration ratio of 6-BA to NAA significantly influenced callus type. When the ratio ranged from 2.0 to 4.0, the proportion of type III callus was highest. Conversely, at ratios ≥ 5.0, the proportion of embryogenic callus decreased, and direct induction of adventitious buds was observed. Considering the analysis of type III callus proportions, the optimal callus induction medium for *E. ulmoides* hypocotyls was determined to be B5 + 1.5 mg/L 6-BA + 0.5 mg/L NAA.

High-quality protoplasts are essential for transformation and functional gene analysis [54]. The plant cell wall primarily comprises cellulose and pectin, with compositions varying across species and even within different organs of the same species [60]. Therefore, the enzymatic method for protoplast isolation must be tailored to the specific cell wall components of the plant material in question [23]. The choice and concentration of cell wall-degrading enzymes, along with the enzymolysis duration, are pivotal in optimising protoplast yield and viability [61]. In previous research, a complex enzyme mix (2.5% cellulase R-10, 0.6% macerozyme R-10, 2.5% pectinase, and 0.5% hemicellulase) yielded 1.13 × 10^6^ protoplasts/g FW from young *E. ulmoides* stems [62]. However, our study demonstrates that a simpler combination of 1.5% cellulase R-10 and 1.0% macerozyme R-10 achieves a higher yield of 1.82 × 10^6^ protoplasts/g FW. This could be attributed to the more intricate cell wall structure of young *E. ulmoides* stems compared to embryogenic callus. The results suggest that callus is a more suitable material for protoplast isolation, aligning with Russell’s results [63]. Numerous studies indicate that increasing enzyme concentration boosts protoplast yield but diminishes viability [62,64]. Interestingly, our research found that the cellulase R-10 concentration had no significant effect on protoplast viability, while higher macerozyme R-10 concentrations reduced yield but enhanced viability. This may occur because increased macerozyme R-10 concentrations improve cell wall degradation efficiency but also stress cells, causing fragile ones to rupture, leaving behind more resilient protoplasts. Enzymolysis duration varies significantly among plant species and tissues. For instance, isolating protoplasts from sweet cherry pulp takes 18 h [65], while it takes 15 h for extraction from strawberry leaves [25] and 10 h for extraction from young *E.* *ulmoides* stems [62]. In contrast, in our study, only 4 h were required for extraction from the embryogenic callus of *E. ulmoides*, similar to the 3–4 h needed for *C. lanceolata* callus [66]. This efficiency is likely due to the thinner cell walls of callus, which degrade more readily. A shorter enzymolysis period generally prevents protoplast degradation, facilitating their subsequent use [66]. The protoplast isolation system developed in this study, with its brief enzymolysis time, ensures the protoplasts remain in a good physiological state, providing a solid foundation for further studies on gene function, protein subcellular localisation, and protein interaction.

When protoplasts are devoid of a cell wall, they can contract, expand, or even rupture due to their inability to maintain isobaric conditions with the external environment [62]. To counteract this, an appropriate concentration of D-mannitol is typically added to the enzymatic solution to balance the osmotic pressure. Studies have indicated that a 0.4 M concentration of D-mannitol is optimal for isolating protoplasts in various plants, including poplar, *C. lanceolata*, and Camellia sinensis [67]. However, both this study and Hu et al. [62] identified that a 0.6 M concentration is necessary for *E. ulmoides*. This higher requirement is likely due to the elevated solute concentration in *E. ulmoides* cells, which contain numerous macromolecular substances like *Eu*-rubber, necessitating a greater concentration of D-mannitol to achieve osmotic balance.

PEG4000 facilitates the uptake of exogenous DNA into protoplasts by altering the physicochemical properties of the cell membrane surface and enhancing membrane permeability. However, its transformation efficiency is influenced by concentration, plasmid dosage, and transformation duration [12,26]. Our study identified that a 40% concentration of PEG4000 optimally supports transformation. Both excessive and insufficient concentrations markedly decrease efficiency, consistent with findings in other plant species [68]. This suggests that approximately 40% PEG4000 strikes an optimal balance between high membrane permeability and low cytotoxicity across various plants, including *E. ulmoides*. The results of this study indicate that a transformation rate of 53.23% can be achieved with just 10 µg of plasmid for 20 min. This plasmid quantity is notably lower than that required for other species, such as *S. spontaneum* [13], *Camellia yubsienensis* [69], and *P. aegyptiaca* [7]. Additionally, both the plasmid dosage and transformation rate surpass those observed in the transient transformation of protoplasts isolated from young stems [62]. This enhanced efficiency may be attributed to the higher membrane permeability of protoplasts derived from *E. ulmoides* embryogenic callus at 40% PEG4000. These results underscore the advantages of the protoplasts obtained through this experimental approach for further research in *E. ulmoides*.

## 4. Materials and Methods

### 4.1. Plant Materials and Culture Conditions

The mature seeds come from the well-grown *E. ulmoides* trees on the campus of Northwest A&F University in Yangling City, Shaanxi Province, China. Mature seeds were removed from their shells and soaked in water for 8 h. They were then washed with a detergent containing a surfactant and thoroughly rinsed under running tap water for 30 min to ensure all residues were eliminated. Subsequently, the seeds were placed on a sterilised ultra-clean table, soaked in 75% (*v*/*v*) ethanol for 30 s, and rinsed 1–2 times with sterile water. Following this, they were immersed in 10% NaClO (*v*/*v*) for 20 min and rinsed 4–5 times with sterile water. The sterilised seeds were trimmed at both ends and inoculated onto Murashige and Skoog (MS) (Coolaber, Beijing, China) [70] medium containing 10 mg/L Gibberellin acid (GA_3_) (Macklin, Shanghai, China), with a pH of 5.8 ± 0.2. The cultures were maintained at a temperature of 24 ± 2 °C under a 16 h/8 h light-dark cycle. After 15 days, the sterile seedlings of *E. ulmoides* reached a height of 5–8 cm, with hypocotyls measuring 3–5 cm.

### 4.2. Callus Induction and Embryogenic Callus Identification

The hypocotyls were sliced into 5–8 mm and inoculated onto B5 medium (Solarbio, Beijing, China) with varying concentrations of 6-Benzyl Aminopurine (6-BA) and α-Naphthaleneacetic acid (NAA) (both from Solarbio, China). A two-factor, five-level, full factorial experimental design was employed, 6-BA and NAA concentrations set at 0.2 mg/L, 0.5 mg/L, 1.0 mg/L, 1.5 mg/L, and 2.0 mg/L, respectively, resulting in 25 distinct treatment combinations (Table 1). Each treatment included 30 explants and was replicated three times. The culture conditions for explants were the same as those for sterile seedlings. After 30 days, the induction rate of callus was calculated by (Number of explants with callus/Total number of explants) × 100%. The state of callus was observed using a light microscope (OLYMPUS BX61, Tokyo, Japan) on the same day. The contribution rate of different callus types was calculated by (Number of callus with specific type/Total number of callus) × 100%. The proportion of different callus types was calculated under different ratios of 6-BA to NAA by (Number of callus with specific type and ratio/Total number of callus with specific ratio) × 100%.

The paraffin sectioning method, adapted from Li’s approach [71], was utilised to distinguish between embryogenic and non-embryogenic callus. In brief, the callus was fixed in a 5% Formalin-Aceto-Alcohol (FAA) (90 mL 50% alcohol, 5 mL acetic acid, 5 mL formaldehyde) solution for 24 h, dehydrated through a gradient ethanol series, embedded in paraffin, and then sliced into 8 μm-thick sections, stained with safranin-fast green, sealed with neutral gum, and observed under a light microscope (OLYMPUS BX61, Tokyo, Japan). The cytological characteristics of embryogenic callus were observed with reference to the description by Qin [72].

### 4.3. Protoplast Isolation and Purification

The isolation and purification of protoplasts were optimised according to the protocols of Yang [40] and He [46]. Briefly, 1.5 g of embryogenic callus was sliced into 1–2 mm particles and swiftly transferred into 15 mL of an enzyme solution (pH = 5.7) containing 0.1% Bovine serum albumin (BSA) (Solarbio, China), 10 mM CaCl_2_, 0.1% 2-(N-morpholino)ethanesulfonic acid monohydrate (MES) (Macklin, China), 0.6 M D-mannitol (Coolaber, China) and varying concentrations of cellulase R-10 and macerozyme R-10 (both from YAKULT, Tokyo, Japan). The mixture was then subjected to enzymatic treatment at 25 °C and 70 rpm in darkness for 4–10 h to dissolve the cell wall and release the protoplasts. To determine the optimal conditions for protoplast isolation, A three-factor and four-level orthogonal design was employed, and each treatment was performed in triplicate to ensure reproducibility. This design examined the concentrations of cellulase R-10 (0.5, 1.0, 1.5, 2.0%) and macerozyme R-10 (0.6, 0.8, 1.0, 1.2%), alongside the enzymatic hydrolysis duration (4, 6, 8, 10 h), resulting in sixteen distinct treatments. Following the identification of the optimal enzyme concentrations and digestion time, various D-mannitol concentrations (0.2, 0.3, 0.4, 0.5, 0.6, 0.7, 0.8 M) were tested to ensure the ideal osmotic pressure was maintained, and each treatment was performed in triplicate.

For purification, the hydrolysate was filtered twice through a 70 μm cell strainer to remove larger tissue. Subsequently, centrifugation was conducted at varying speeds (400, 600, 800, 1000, and 1200 rpm) for 5 min at room temperature to investigate the enrichment effect of centrifugal force on protoplasts. Each treatment was performed in triplicate. Then, the obtained protoplasts were resuspended in 5 mL of W5 solution (comprising 154 mM NaCl, 125 mM CaCl_2_, 5 mM KCl, 5 mM glucose, 2 mM MES) and washed three times. Finally, the protoplasts were resuspended in 1 mL of MMG solution (containing 15 mM MgCl_2_, 4 mM MES, 0.6 M D-mannitol, pH = 5.7) and incubated on ice for 30 min to assess the yield and viability of the protoplasts and perform transient transformation.

### 4.4. Protoplast Yield and Viability Assessment

The yield of protoplasts was assessed using a haemocytometer under a light microscope (Olympus BX61, Tokyo, Japan,). Protoplasts were stained with 0.1% (*w*/*v*) fluorescein diacetate (FDA) (Solarbio, China) in darkness for 5 min, after which their viability was examined using a fluorescence microscope (Carl Zeiss, Gottingen, Germany, Axio Imager M2), and FDA reacted with living cells to fluoresce green under excitation light of 488 nm. Protoplasts exhibiting green fluorescence were deemed viable. The calculations for protoplast yield and viability were as follows: Protoplast yield (protoplasts/g FW) = total number of protoplasts obtained/fresh weight of calls; Protoplast viability (%) = (number of the fluorescent protoplasts/total number of protoplasts) × 100%. Each treatment was conducted in triplicate.

### 4.5. Protoplast Transformation

Polyethylene glycol (PEG)-mediated protoplast transfection was carried out as described by Zhang [37] and Yang [40] with slight modifications. In brief, freshly isolated protoplasts were suspended in MMG solution to reach a final concentration of 1 × 10^5^ protoplasts/mL. The purified pCAMBIA3301-35S-eGFP plasmid was introduced into a 2 mL round-bottom tube containing 110 μL of PEG buffer (containing 0.6 M D-mannitol and 0.1 M CaCl_2_) and 100 μL of the protoplast suspension, then gently mixed. After infection for various durations at 25 °C in the dark, we added 420 μL of W5 solution to terminate the transformation. Then, the mixture was centrifuged at 1000 rpm for 5 min, and the supernatant and protoplasts were washed twice with W5 solution.

To optimise transformation conditions, transient transformation experiments were conducted using varying concentrations of plasmid DNA (5 μg, 10 μg, 15 μg, 20 μg) with 40% PEG4000 and an incubation period of 20 min. Additionally, different PEG4000 concentrations (20%, 30%, 40%, 50%) were tested, maintaining a 20 min incubation and using 10 μg of plasmid. For the combination of 40% PEG4000 and 10 μg plasmid DNA, incubation times were varied at 5, 10, 15, 20, 25, and 30 min. The transformed protoplasts were cultured in darkness at 25 °C for 12 h before being examined under a fluorescence microscope. Green fluorescent protein (GFP) expression in the transfected protoplasts was observed using a fluorescence microscope with an excitation wavelength of 488 nm. Transformation efficiency was calculated using the formula: Transformation efficiency (%) = (number of fluorescent protoplasts/total number of protoplasts) × 100%. Each combination of conditions was performed in three independent experiments, observing five fields per replicate.

### 4.6. Detection of Transfected Protoplasts by PCR

Total genomic DNA from transfected and untransfected (control) protoplasts was extracted using the DNAsecure Plant Kit (TIANGEN, Beijing, China). *M13* universal primers and enhanced GFP (*eGFP*) and neomycin phosphotransferase (*NPTII*) specific primers were used to detect the presence of pCAMBIA3301*-35S-eGFP* in the transfected protoplasts but not in the untransfected ones (Table 2). The PCR procedure was pre-denatured at 94 °C for 3 min; 35 cycles of denaturation at 94 °C for 30 s, annealing at 58 °C for 30 s, extended at 72 °C for 1 min; and extended at 72 °C for 5 min. Subsequently, PCR products were analysed by 1% (*w*/*v*) agarose gel electrophoresis.

### 4.7. Data Analysis

All quantitative data are presented as the mean ± standard deviation from at least three independent replicates. The statistical analysis was conducted using IBM SPSS Statistics Version 21.0 (SPSS Inc., Chicago, IL, USA). Data from the two-factor full factorial experiment (callus induction) were analysed by two-way ANOVA to assess main and interaction effects, followed by simple effects analysis and Fisher’s Least Significant Difference (LSD) test where appropriate. For the orthogonal experiments (protoplast isolation), significant factors were identified by ANOVA, and optimal conditions were determined via range analysis (R). All single-factor optimisation experiments (including those for mannitol concentration, centrifugation speed, and transient transformation conditions) were analysed by one-way ANOVA with LSD post hoc tests. Prior to all ANOVAs, assumptions of normality (Shapiro–Wilk test) and homogeneity of variances (Levene’s test) were verified. Statistical significance was defined as *p* < 0.05.

## 5. Conclusions

In this study, we established an efficient and reproducible system for embryogenic callus induction, protoplast isolation, and PEG-mediated transient transformation in *E. ulmoides.* Hypocotyls from 15-day-old sterile seedlings of *E. ulmoides* served as explants and were cultured on B5 solid medium supplemented with 1.5 mg/L 6-BA, 0.5 mg/L NAA and 30 g/L sucrose for 30 d, resulting in a contribution rate of 86.3% for embryogenic callus. The embryogenic callus was digested in an enzyme solution containing 1.5% cellulase R-10 and 1.0% macrozyme R-10 under 0.6 M D-Mannitol osmotic pressure for 4 h, followed by collection through centrifugation at 1000 rpm. High-quality protoplasts were obtained, yielding 1.82 × 10^6^ protoplasts/g FW. When incubated with 10 μg of plasmid and 40% PEG4000 for 20 min, the transient transformation efficiency reached 53.23%. Furthermore, the developed method presents a convenient technology for applications such as protein subcellular localisation, protein-DNA interaction, protein–protein interaction, genome editing, and various other molecular biology research endeavours in *E. ulmoides*.

## Figures and Tables

**Table 1:** Impact of various 6-BA and NAA concentration combinations on callus induction rate in *E. ulmoides*.

Treatment	6-BA (mg/L)	NAA (mg/L)	Induction Rate (%)	Primary Types
T1	0.2	0.2	93.33 ± 1.44 cde	Type II
T2	0.2	0.5	97.50 ± 0.00 ab	Type I
T3	0.2	1.0	97.50 ± 0.00 ab	Type I
T4	0.2	1.5	94.17 ± 1.44 bcd	Type I
T5	0.2	2.0	100.00 ± 0.00 a	Type I
T6	0.5	0.2	95.00 ± 0.00 bc	Type III
T7	0.5	0.5	97.50 ± 0.00 ab	Type II
T8	0.5	1.0	97.50 ± 0.00 ab	Type I
T9	0.5	1.5	100.00 ± 0.00 a	Type I
T10	0.5	2.0	96.67 ± 1.44 abc	Type I
T11	1.0	0.2	93.33 ± 1.44 cde	Type III
T12	1.0	0.5	90.00 ± 4.33 efg	Type III
T13	1.0	1.0	86.67 ± 5.77 gh	Type II
T14	1.0	1.5	85.83 ± 2.89 h	Type I
T15	1.0	2.0	81.67 ± 1.44 i	Type I
T16	1.5	0.2	95.00 ± 2.50 bc	Type II
T17	1.5	0.5	97.50 ± 0.00 ab	Type III
T18	1.5	1.0	82.50 ± 0.00 i	Type II
T19	1.5	1.5	94.17 ± 1.44 bcd	Type II
T20	1.5	2.0	97.50 ± 0.00 ab	Type I
T21	2.0	0.2	88.33 ± 3.82 fgh	Type II
T22	2.0	0.5	94.17 ± 1.44 bcd	Type III
T23	2.0	1.0	80.00 ± 0.00 i	Type III
T24	2.0	1.5	93.33 ± 1.44 cde	Type II
T25	2.0	2.0	90.83 ± 1.44 def	Type II

Note: Different lowercase letters indicate significant differences (*p* < 0.05).

**Table 2:** Primer information for detecting the pCAMBIA3301-35S-eGFP plasmid [64].

Gene	Forward (5′-3′)	Reverse (5′-3′)	Tm (°C)
*M13*	CAGGAAACAGCTATGAC	GTAAAACGACGGCCAGT	58
*eGFP*	GGTACCCGGGGATCCTCT	GAAAGCTCTGCAGGAATTCGATT	58
*NPTII*	AACTCACGTTAAGGGATTTTGGTCAT	TCTTGGGGTATCTTTAAATACTGTAGAAAAGAGGA	58

## Data Availability

All data are included in the present study.
